# Application of Proteomics to Soft Tissue Sarcomas

**DOI:** 10.1155/2012/876401

**Published:** 2012-06-19

**Authors:** Tadashi Kondo, Daisuke Kubota, Akira Kawai

**Affiliations:** ^1^Division of Pharmacoproteomics, National Cancer Center Research Institute, 5-1-1 Tsukiji, Chuo-ku, Tokyo 104-0045, Japan; ^2^Division of Musculoskeletal Oncology, National Cancer Center Hospital, 5-1-1 Tsukiji, Chuo-ku, Tokyo 104-0045, Japan

## Abstract

Soft tissue sarcomas are rare and account for less than 1% of all malignant cancers. Other than development of intensive therapies, the clinical outcome of patients with soft tissue sarcoma remains very poor, particularly when diagnosed at a late stage. Unique mutations have been associated with certain soft tissue sarcomas, but their etiologies remain unknown. The proteome is a functional translation of a genome, which directly regulates the malignant features of tumors. Thus, proteomics is a promising approach for investigating soft tissue sarcomas. Various proteomic approaches and clinical materials have been used to address clinical and biological issues, including biomarker development, molecular target identification, and study of disease mechanisms. Several cancer-associated proteins have been identified using conventional technologies such as 2D-PAGE, mass spectrometry, and array technology. The functional backgrounds of proteins identified were assessed extensively using *in vitro* experiments, thus supporting expression analysis. These observations demonstrate the applicability of proteomics to soft tissue sarcoma studies. However, the sample size in each study was insufficient to allow conclusive results. Given the low frequency of soft tissue sarcomas, multi-institutional collaborations are required to validate the results of proteomic approaches.

## 1. General Background of Soft Tissue Sarcomas

Soft tissue sarcomas are defined as malignancies that originate from nonepithelial extraskeletal tissues, excluding the reticuloendothelial system, glia, and supporting tissue of parenchymal organs [1].Thus, soft tissue sarcomas may occur anywhere in the body including fibrous tissues, adipose tissues, muscles, vessels, and peripheral nerves. Thus, soft tissue sarcomas are a highly heterogeneous tumor group that is classified histogenetically. Clinically, soft tissue sarcomas range from curable tumors to those causing death via metastasis and recurrence. Thus, histological classification is accompanied by grading and staging information, which depends on clinical and pathological observations, to assess the degree of malignancy, predict prognosis, and evaluate possible therapies.

Genetic alterations such as recurrent chromosomal translocations and genetic deregulations have been reported in soft tissue sarcomas. For example, chimeric genes have been reported in Ewing sarcoma, alveolar rhabdomyosarcoma (RMS), desmoplastic small round cell tumor, clear cell sarcoma (CCS), myxoid/round cell liposarcoma, myxoid chondrosarcoma, synovial sarcoma, alveolar soft part sarcoma (ASPS), and fibromyxoid sarcoma [1]. Some soft tissue sarcomas depend on genetic deregulation of kinase signaling pathways, including inflammatory myofibroblastic tumor, congenital fibrosarcoma, dermatofibrosarcoma protuberans, giant cell tumor of the tendon sheath, and gastrointestinal stromal tumor (GIST) [1]. Unique genetic alterations have diagnostic and prognostic value and provide a clue for therapeutic targets, for example, a small molecule directed against tyrosine kinase reduced metastasis after surgical resection in GIST [2]. However, the effects of common genetic aberrations on the malignant behavior of tumor cells is not completely understood, and typical molecular backgrounds have not been reported for most soft tissue sarcomas.

Soft tissue sarcomas are relatively rare compared with other malignancies and constitute less than 1% of all malignant cancers [3]. Development of diagnostic and prognostic modalities, identification of therapeutic targets, and understanding disease mechanisms are the main research priorities in soft tissue sarcoma studies. These studies generally demand the use of a range of clinical materials with different clinicopathological data. However, the low incidence and extensive heterogeneity of tissue types indicates that it is challenging to obtain sufficient volume of clinical materials for conclusive results in expression studies and validation purposes.

## 2. Application of Proteomics to Soft Tissue Sarcomas

The proteome is a functional translation of the genome, which directly regulates the phenotypes of tumor cells. Thus, proteomic approaches could make significant contributions to diagnosis and classification of soft tissue sarcomas. Comprehensive studies at DNA and mRNA levels have provided several valuable findings. For example, studies of changes in gene copy number using array-based CGH have demonstrated relationships among malignant fibrous histiocytoma (MFH), leiomyosarcoma, and liposarcoma [4, 5]. Expression profiling of 5520 genes at the mRNA level using microarrays has revealed distinct molecular signatures for 41 sarcomas, thereby providing new markers with potential diagnostic value [6]. Furthermore, expression microarrays have identified robust, novel sarcoma-specific immunohistochemical markers [7–10]. These results are expected to lead to novel therapeutic modalities in the near future. From another viewpoint, many lines of evidence have suggested a disparity between mRNA and protein expression data, although mRNA expression studies appear to be promising [11, 12]. These observations have recently been supported by a quantitative mRNA and protein expression study that revealed that protein abundance was predominantly controlled at the translation level [13]. Furthermore, posttranslational modifications and formation of protein complexes, which are often aberrantly regulated in tumor cells and result in tumor formation, cannot be predicted by gene copy number and at the mRNA level [14]. Thus, proteomics may be an indispensable approach for understanding the molecular backgrounds of soft tissue sarcomas. The following section describes how proteomic approaches have been applied to soft tissue sarcomas.

### 2.1. Proteomic Backgrounds of Different Histological Subtypes

Soft tissue sarcomas are classified according to their source tissues and histological appearance is clearly distinct in typical cases. Unique genetic alterations have been reported in certain tumors, but molecular histological backgrounds have not been defined for most tumors and differential diagnosis is often difficult [15]. To assess the proteome backgrounds of soft tissue sarcomas, Suehara et al. compared primary tumor tissues of seven types of soft tissue sarcomas, including CCS, myxoid liposarcoma, GIST, malignant peripheral nerve sheath tumor (MPNST), MFH, and leiomyosarcoma [16]. A global protein expression study using two-dimensional difference gel electrophoresis (2D-DIGE) identified 67 proteins that distinguished tumors with different histologies. The protein expression data indicated that 10 proteins, including tropomyosin subtypes, discriminated pleomorphic leiomyosarcoma from the conventional one. The molecular signatures of histological subtypes were investigated extensively at the mRNA level using DNA microarrays [6]. However, results of protein and mRNA studies did not overlap in the different patient populations, probably because of the discrepancy between mRNAs and proteins as well as the different scope of proteomics and transcriptomics. A comprehensive understanding of the molecular backgrounds of soft tissue sarcomas with different histologies will demand the integration of multiple proteomic studies using a common tissue sample set. 2D-DIGE covers a limited proportion of the proteome, hence, it would be beneficial examining other proteomic modalities to detect practical biomarkers for differential diagnosis and validate the reported proteins and mRNAs.

### 2.2. Liposarcoma

Liposarcoma is one of the most common soft tissue sarcomas in adults [17]. Liposarcoma includes numerous subtypes with clinical courses ranging from localizing neoplasms to high-grade sarcomas with metastatic potential [18].

To develop diagnostic biomarkers, Cervi et al. conducted proteomic studies using mice with xenografts from liposarcoma, osteosarcoma, and adenocarcinoma cell lines [19]. Based on the hypothesis that platelet protein content may indicate the presence of a tumor, platelet-rich plasma samples were compared in nontumor-bearing and xenograft mice. Proteomic studies using surface-enhanced laser desorption/ionization time-of-flight mass spectrometry (SELDI-ToF MS) revealed that platelet factor-4 (PF-4) was upregulated in platelets of tumor-bearing mice. Previously, it has been reported that PF-4 is a suppressor of angiogenesis and tumor growth [20, 21]. Increase in PF-4 levels in platelets was observed in both angiogenic and nonangiogenic xenografts of liposarcoma, whereas it was upregulated only in angiogenic xenografts of breast cancer and osteosarcoma. Moreover, higher PF-4 levels in the platelets were observed as early as 19 days after xenograft implantation. These observations suggest that PF-4 may be useful for early tumor detection in liposarcoma.

An unique aspect of this study was that the platelet proteome was examined for biomarkers. Platelets may sequester angiogenesis-regulating proteins and protect them from plasma proteolytic enzymes. This study was a good example of how focused proteomics can identify biomarker candidates that cannot be detected by other approaches. This study should be followed by the functional assessment of PF-4 levels in platelets of patients with liposarcoma and extensive validation of clinical materials.

### 2.3. Gastrointestinal Stromal Tumor (GIST)

GIST is the most common mesenchymal malignancy of the gastrointestinal tract [22, 23]. It emerges from the interstitial cells of Cajal and is characterized by the presence of gain-of-function mutations in c-kit or platelet-derived growth factor receptor [24]. These mutations result in constitutive activation of signaling pathways downstream of receptor type kinases. Recently, a small molecule tyrosine kinase inhibitor, imatinib mesylate (ST12571, Novartis), was reported to be highly effective in treatment of GIST [25–27]. Although the effects of imatinib mesylate are obvious, only a limited number of patients may benefit from treatment because the proportion of highly malignant or high-risk GIST is only 20%–35%, while 60%–80% of GIST patients can be cured by surgical resection [22, 23]. Therefore, biomarkers to assess the malignant potential of GIST and predict postsurgery metastasis are required to optimize therapeutic strategies.

Suehara et al. investigated the proteome of primary tumor tissue of GIST to develop a biomarker indicative of imatinib mesylate benefit [28]. They compared the protein content in patients with metastasis within 1 year of surgery with that in patients without metastasis for at least 2 years after surgery. Proteomic study with 2D-DIGE identified 43 proteins with significantly different expression levels in the two groups. Among these, they further focused on the protein pfetin. Pfetin expression was significantly higher in patients with a better prognosis than that in patients with a poor prognosis. Pfetin was originally cloned as a gene with specific expression in the fetal cochlea [29], while it was recently identified as a component of the GABAB receptor [30]. However, pfetin was not associated with any type of malignancy before this study. The prognostic utility of pfetin was validated in 210 GIST cases by immunohistochemistry. Further validation studies were conducted for 140 cases in two other hospitals [31, 32].

Kikuta et al. also searched for prognostic biomarkers of GIST using a large format 2D-DIGE that generated up to 5,000 protein spots [33]. In a comparison of primary tumor tissues of GIST from patients with poor and good prognoses, they identified ATP-dependent RNA helicase DDX39 (DDX39) as a biomarker of a worse prognosis [34]. DDX39 was originally reported as a growth-associated RNA helicase, and its overexpression has been observed in lung squamous cell carcinoma [35]. DDX39 promoted colony formation, which probably contributed to telomere elongation [36, 37]. The prognostic value of DDX39 has been confirmed in an additional 72 cases from another hospital by immunohistochemistry.

The malignant behavior of GIST was significantly associated with its anatomical site of origin, that is, the clinical course of small intestinal GISTs was more aggressive than that of gastric GISTs [38, 39]. Suehara et al. compared stomach and intestinal GISTs using 2D-DIGE and identified cancer-associated proteins with differential expression depending on their site of origin [40]. These included prohibitin, pigment epithelium-derived factor, and alpha-actinin 4. 35% of the identified proteins had concordant expression at the mRNA level.

These three studies were aimed at clinical applications of proteomics, and they successfully identified candidate biomarkers. It is noteworthy that a multi-institutional validation using immunohistochemistry was successfully achieved for the determination of the prognostic value of pfetin and DDX39. In addition to interinstitutional collaboration, the use of a specific antibody and a conventional examination method such as immunohistochemistry may be the key to a successful validation study.

### 2.4. Leiomyosarcoma

Leiomyosarcoma is a smooth muscle malignancy of the uterus, stomach, small intestine, retroperitoneum, and extremities that comprises 5%–10% of soft tissue sarcomas [41]. Leiomyosarcoma is divided into site-related subgroups such as leiomyosarcoma of the retroperitoneum/abdominal cavity, leiomyosarcoma of somatic soft tissue, and cutaneous leiomyosarcoma. They have significantly different responses to treatment and a broad range of prognoses, but molecular backgrounds that explain the various clinical behaviors have not yet been determined.

Yang et al. investigated proteins specific to leiomyosarcoma [42]. They compared primary tumor tissues of leiomyosarcoma with those of GIST. These two soft tissue sarcomas are the most common mesenchymal tumors and share remarkably similar phenotypic features although they are molecularly and clinically distinct [43]. It is important to separate leiomyosarcoma from GIST because their treatment and prognosis differ significantly. Protein expression of primary tumor tissues of leiomyosarcoma and GIST were profiled using a reverse-phase protein lysate array (RPA). Unique expression of E-cadherin, a marker of epithelial cells, was observed in leiomyosarcoma. A tissue microarray (TMA) study localized E-cadherin in tumor cells and showed that patients with E-cadherin-positive primary tumors had a better prognosis. E-cadherin is considered to be a marker of mesenchymal to epithelial reverting transition (MErT) during metastasis [44]. A transcriptome study indicated the presence of other epithelial marker genes in leiomyosarcoma, supporting the hypothesis that MErT is a novel phenotype in leiomyosarcoma. An *in vitro* assay indicated that expression of E-cadherin and other epithelial marker genes was regulated by Slug. Taken together, this study was the first to demonstrate the MErT phenotype in leiomyosarcoma, which was associated with a beneficial clinical prognosis.

To examine the mechanisms of tumorigenesis in leiomyosarcoma, Dhingra et al. performed TMA of leiomyosarcoma in the uterus and normal myometria and focused on the signaling pathway of mammalian target of rapamycin (mTOR) and phospholipase D (PLD) [45]. This expression study identified constitutive activation of the mTORC2 pathway as well as overexpression of PLD1 and one of its downstream effectors. These observations may suggest the utility of the mTOR-PLD signaling pathway as a therapeutic target in leiomyosarcoma.

RPA facilitates high throughput and quantitative protein expression studies. It does not identify the cellular localization of proteins, and hence, complementary TMA application be useful for interpreting RPA results. Antibody-based proteomics, such as RPA and TMA, are based on *a priori* knowledge of cancer biology during antibody selection, and hence, the functional interpretations of results may be easier than when proteins are randomly observed by 2D-PAGE and MS. Recent studies have identified common aberrations in tyrosine kinases in leiomyosarcoma and GIST [46] as well as molecular subtypes in leiomyosarcoma [47]. This novel knowledge of disease backgrounds may facilitate pathway-wide or molecular group-wide analysis by antibody-based proteomics, such as RPA and TMA, in leiomyosarcoma. However, antibody-based proteomics is limited by the availability of antibodies although the transcriptome approach may compensate for such drawbacks.

### 2.5. Rhabdomyosarcoma (RMS)

RMS is the most common soft tissue sarcoma in children [48, 49], with an overall survival rate in all patients with childhood RMS of 60%–70% [50, 51]. RMS is histologially categorized into two major subtypes—alveolar RMS (A-RMS) and embryonal RMS (E-RMS). A-RMS is characterized by the presence of unique fusion genes, such as PAX3-FOXO1 (P3F) and PAX7-FOXO1, which have more aggressive clinical phenotypes than E-RMS. The detection of P3F has diagnostic value, but a subset of A-RMS does not express P3F, and the functional roles of P3F in the clinical behavior of A-RMS has not been clarified. A detailed understanding of the biological significance of P3F and biomarkers that identify patients who are destined to fail with standard therapy are in great demand.

To examine the pathogenesis of A-RMS, Pressey et al. investigated the effects of P3F on cells [52]. Murine fibroblast cells coexpressing P3F and simian virus 40 large-T antigen exhibited malignant features such as anchorage-independent growth and colony formation. Whole cell lysates and conditioned medium from transformed cells were examined by 2D-DIGE. Of the 1335 proteins observed in the cell lysate, 93 had statistically different expression after transformation, while MS identified the corresponding proteins. Overexpression of valosin-containing protein, one of the identified gene products, was confirmed in A-RMS by immunohistochemical analysis. Proteins were also examined in conditioned medium of the transformed cells by 2D-DIGE. Of the 726 proteins observed, 39 had statistically different expression after transformation. Although the details of the identified proteins were not investigated in this study and external validation was not attempted, these observations indicate the utility of the proteomic approach for capturing changes caused by the unique fusion gene, P3F.

Petricoin et al. conducted a phosphoproteomic network analysis of RMS cells with the aim of developing prognostic biomarkers [53]. Laser microdissected RMS cells were examined by RPA and antibodies against proteins in the Akt and mTOR pathways. The status of the signal transduction pathway was evaluated using antibodies against phosphorylated proteins. Specific phosphorylated forms of Akt, eIF4G, 4EBP1, and p70S6 were associated with survival of patients with RMS. Novel IRS-1 cell signaling pathway linkages in RMS were proposed based on these data. Treatment with a rapamycin analogue, an inhibitor of the mTOR pathway, considerably reduced the growth of RMS xenografts, suggesting that the IRS-1/Akt/mTOR network may have a key role in tumor progression.

These two studies employed two different proteomic approaches, that is, separation-based and antibody-based proteomics. Separation-based proteomics accessed the proteome in a random manner, which may bring unexpected findings such as the induction of the valosin-containing protein by P3F. However, antibody-based proteomics is based on existing knowledge of cancer biology, which is necessarily focused on a limited number of target proteins. The effects of P3F were not predicted before experiments, and hence, the separation-based approach may be an effective strategy. In contrast, we already know that aberrant regulation of signal transduction pathways has a key role in the progression of RMS; therefore, antibody-based proteomics should be considered initially. There is no dominant modality in proteomics and it is important to select the best approach after considering the research goal.

### 2.6. Angiosarcoma

Angiosarcoma is included within the broad category of vascular tumors with aggressive clinical behaviors. A proteomic study of angiosarcoma has only been reported in dogs. Visceral angiosarcoma occurs frequently in large breed dogs with a prevalence of 0.3%–2.0% of all tumors [54, 55]. In visceral angiosarcoma, a local invasion and distant metastasis occur early and diseased dogs are usually taken to hospitals during the late stages of disease, when serious hemorrhage after tumor rupture causes acute anemia or cardiac tamponade [56]. A diagnosis of visceral angiosarcoma usually requires invasive examination, and biomarkers that allow a noninvasive diagnosis are required before early surgical intervention.

Kirby et al. investigated the serum proteome of angiosarcoma in dogs to identify diagnostic biomarkers [57]. Using 2D-DIGE, a truncated form of collagen XXVII was identified that had higher expression in dogs with angiosarcoma. The serum level of truncated collagen XXVII diminished after surgical resection and continued to decrease during chemotherapy. The level of truncated collagen XXVII increased when there was evidence of recurrence. These observations suggested the use of the truncated form of collagen XXVII as a diagnostic biomarker.

Angiosarcoma is a highly vascularized neoplasm, and thus, it was expected that proteins derived from the tumor or tumor-surrounding tissues would be found in blood circulation. The high vascularity of angiosarcoma is common in dogs and human [58]. Thus, it would be worthwhile to apply a similar proteomic approach to human angiosarcoma to determine whether the expression of serum collagen XXVII has a similar diagnostic value in human angiosarcoma.

### 2.7. Kaposi Sarcoma (KS)

KS is an angioproliferative disorder of the vascular endothelium. There are four clinical variants, each with its own natural history, site of predilection, and prognosis [59]. The clinical course of KS varies from an indolent course in the classic variant to a progressive and fatal course in the epidemic variant [60]. KS pathogenesis involves infection with KS-associated herpesvirus (KSHV). KSHV induces angiogenic and inflammatory cytokines as well as gene products implicated in angiogenesis that directly contribute to KS pathogenesis [61]. In addition to KS, KSHV is an etiological agent that is associated with development of other malignancies including primary effusion lymphoma and multicentric Castleman's disease, suggesting the versatile carcinogenic properties of KSHV. KSHV should be studied in detail to aid our understanding of KS pathogenesis, develop biomarkers, and discover therapeutic targets.

Zhu et al. identified proteins associated with KSHV virions when investigating KSHV primary infection, virion formation, and egress [62]. Virion particles were purified from the supernatant of KSHV-infected cells, and virion proteins were extracted and separated by SDS-PAGE. The proteins were recovered as tryptic digests and subjected to LC-MS/MS. Overall, 24 KSHV virion proteins were identified, and their expression and cellular localization were confirmed by specific antibodies.

Associations of proteins produced by KSHV and host cell proteins were individually investigated using proteomic approaches. Jong et al. studied proteins binding to viral protein kinases (vPKs) [63]. Proteins associated with glutathione-S-transferase (GST) fusion vPKs were purified and separated by SDS-PAGE. MS identified RNA helicase A as a vPK-interacting cellular protein. Subsequent studies demonstrated that the transactivation ability of RHA in CREB-dependent transcription was regulated by vPKs.

 Latency-associated nuclear antigen (LANA) is a viral protein involved in latent infection and is a multifunctional protein that modulates activation and repression of cellular protein transcription. By interacting with cellular proteins, LANA inhibits apoptosis, transcription, and signal transduction. Kaul et al. investigated proteins associated with LANA [64]. Nuclear extracts of KSHV-positive cells were incubated with N- or C-terminal LANA fused with GST. Proteins binding to GST-fusion LANA were separated by SDS-PAGE and subjected to MS. A total of 53 and 56 proteins were identified based on their association with the amino- and carboxy-terminal domains of LANA, respectively. These included proteins involved in regulation of signaling pathways, cell division, transcription, and cytoskeleton organization. These observations suggested novel cellular functional areas that may be involved in KSHV biology, and further examinations of the identified proteins may contribute to novel clinical applications.

Viruses affect recognition by immune cells by interfering with immunologically relevant host cell proteins such as MHC-I. To identify the targets of viral immune regulation, Bartee et al. investigated host proteins associated with the viral immune modulator K5, which is encoded by KSHV [65]. Stable isotope labeling of amino acids in cell culture (SILAC) was used for quantitative proteomics [66], and membrane proteins were extracted from cells overexpressing K5. ALCAM, BST-2, syntaxin 4, and MHC-I were reproducibly downregulated by K5 in the plasma membrane fraction. The downregulation of ALCAM, BST-2, and syntaxin 4 by K5 were novel findings, which were subsequently confirmed using specific antibodies. Investigation of immunomodulation by K5 via its binding to these proteins will pave the way to a greater understanding of KSHV pathogenesis.

Interactions among proteins produced by KSHV have been investigated in a comprehensive manner. Uetz et al. cloned 89 open reading frames (ORFs) from KSHV and performed a yeast two-hybrid (Y2H) experiment [67]. They identified 123 nonredundant protein-protein interactions, and 50% of these were confirmed by coimmunoprecipitation. Protein data revealed that the viral protein interaction network had distinct features compared with the cellular protein network. To understand capsid formation, tegumentation, and envelopment, Rozen et al. focused on the protein-protein interactions among virion proteins and constructed a virion-wide protein interaction map based on Y2H and coimmunoprecipitation [68].As a result, 37 protein-protein interactions were identified, which included seven interactions between tegument and capsid proteins, 15 among tegument proteins, and 17 between tegument and envelope proteins. The interaction maps of these two studies and subsequent functional validation studies may contribute to the further understanding of KSHV biology.

Zheng et al. studied humoral immune responses to KSHV [69]. Plasma samples from HIV-positive individuals with KS were reacted with KSHV proteins. The antigens that most frequently reacted with patient plasma samples were ORF38, a myristoylated tegument protein, and ORF73, LANA. The envelop glycoprotein K8.1 is the most common antigen used to investigate antibodies against lytic KSHV infection, while K8.1 was also recognized based on HIV-positive KS sera. Plasma samples from HIV-positive patients with lymphoma were examined and found to have distinct immune responses. The clinical utility and pathological significance of the identified antigens ORF38 and ORF73 are of great interest and should be addressed in future studies.

Proteomic studies of KSHV have focused extensively on the KSHV interactome and many hypotheses have been proposed based on this network analysis. In contrast, only a few studies have been performed to examine these hypotheses and results have not been associated with clinical observations. The next challenge is to clarify whether *in vitro* proteome studies can contribute to clinical applications.

### 2.8. Malignant Mesothelioma (MM)

MM is an aggressive malignancy that develops from mesothelial cells lining the pleural, peritoneal, and pericardial cavities [70]. MM is considered to be an almost incurable tumor, with resistance to chemo- and radiotherapy and a median survival rate of 8–18 months [71, 72]. The etiology of MM is associated with prior exposure to asbestos, which was widely used as a construction material throughout the world and the incidence of the disease in increasing.

A persistent inflammatory response initiated by reactive oxygen species, growth factors, and/or various proinflammatory factors such as cytokines and chemokines, may underlie the pathogenesis of asbestos-associated diseases. Based on this hypothesis, Hilegass et al. examined the proteins released by mesothelial cells exposed to crocidolite asbestos to assess the pathogenicity of asbestos against mesothelial cells [73]. A multiplex suspension protein array of 27 cytokines showed that levels of four cytokines increased after cells were exposed to crocidolite asbestos. Parallel experiments using a DNA microarray showed that these asbestos induced changes were not necessarily associated with concomitant changes in transcript levels. These discrepancies may indicate the release of cytokines/growth factors from cells, or post-translational/transcriptional regulation. These observations may contribute to an understanding of the early molecular events caused by asbestos and development of *in vitro* screening tests for asbestos toxicity.

The differential diagnosis of malignant pleural mesothelioma (MPM) from other lung malignancies is important. There are panels of antibodies against MPM markers such as calretinin, podoplanin, cytokeratin 5/6, and WT-1, but the discrimination of MPM from other lung malignancies, particularly lung adenocarcinoma (ADCA), is still challenging [70, 74, 75]. Hosako et al. compared the proteome of MPM with that of malignancies found in the pleura that are included in the differential diagnosis of MPM [76]. Cells of MPM, lung adenocarcinoma, squamous cell carcinoma of the lung, pleomorphic carcinoma of the lung, and synovial sarcoma were recovered and subjected to large format 2D-DIGE. Of the 3875 proteins observed, cathepsin D was overexpressed in lung adenocarcinoma compared with MPM. The frequent overexpression of cathepsin D in ADCA was confirmed in newly enrolled cases of MPM (46 cases) and ADCA (150 cases) by TMA.

Cell surface proteins are considered candidate biomarkers and therapeutic targets. Ziegler et al. compared N-glycosylated proteins on the cell surface of an MPM cell line and the ADCA cell line Calu-3 [77]. The SILAC method was used for quantitative proteomics [66]. The sugar moieties on the cell surface exposed glycoproteins that were labeled using biotin hydrazide; after protein digestion with trypsin, the glycopeptides were enriched with streptavidin-coated beads. The glycosylated peptides were specifically recovered by PNGase F and subjected to LC-MS/MS. This approach detected 100 cell surface N-glycoproteins including the known MPM markers mesothelin and N-cadherin. The unique expression of deglycosylated thy-1/CD90 was confirmed by western blotting and immunohistochemistry. These two studies demonstrated the application of proteomics to tumor tissues in biomarker studies. However, the clinical utility of the biomarker candidates has not been established, and further validation studies will be required before clinical applications can be developed.

The presence of pleural effusions is a hallmark of MPM onset. Analysis of pleural effusions provides a picture of the *in situ* activity of tumor cells on pleural membranes, and proteomics for pleural effusions would be of interest for basic and clinical purposes. Hegmans et al. compared pleural effusions in patients with MPM and patients with effusions due to other causes [78]. SELDI-ToF MS revealed a protein peak with significant sensitivity and specificity for MPM, which was later identified as apolipoprotein C1. This study should be followed with a validation study to determine the practical utility of the fragment of apolipoprotein C1. To assess immunoresponse in patients with MPM, Hegmans et al. examined 80 cytokines in a conditioned medium from MPM cell lines and pleural effusions of the original patients from whom cell lines were established [79]. An antibody-based cytokine array system demonstrated the production of many cytokines in both MPM cell line supernatants and pleural effusions or exclusively in pleural effusions. These included factors related to angiogenesis, leukocyte attraction, and suppression of the antitumoral immune response, suggesting leukocyte recruitment from circulating blood cells. Subsequent immunohistochemistry showed that mesothelioma tissues included inflammatory cells such as leukocytes, macrophages, and natural killer cells. The clinical and biological implications of recruited inflammatory cells are of particular interest because they are often associated with carcinogenesis and prognosis of patients with other malignancies [80].

Exosomes are membrane vesicles with an endosomal origin [81], and those from tumor cells could be used for biomarker development and immunotherapy programs in MPM [82]. Bard et al. examined exosomes in pleural effusions of patients with various lung malignancies including MPM [83]. Exosomes were isolated from pleural effusions by the sucrose gradient method. Exosome proteins were separated by SDS-PAGE and subjected to MALDI-TOF analysis for protein identification. MS identified a high amount of immunoglobulins and complement factors. In addition to reported exosome proteins such as MHC-I molecules, they detected cytoskeletal proteins, signal transduction proteins, and novel exosome proteins, which were associated with membrane trafficking, cell growth, differentiation, organization of the extracellular matrix, or cell-matrix interaction. Exosomes in pleural effusions may not be secreted by MPM alone but also by mesothelial cells or cells of hematopoietic origin that are present in the fluid. To discriminate exosomes with an MPM origin, Hegmans et al. examined exosomes in the culture supernatant of MPM cell lines using the same approach, that is, SDS-PAGE separation, followed by MALDI-TOF analysis [84]. The 38 proteins identified included MHC-I molecules, cytoskeletal proteins, heat shock proteins, and proteins involved in the organization of membrane traffic, the extracellular matrix, or cell-matrix interaction. A precursor of developmental endothelial locus-1 was also identified as a novel exosome protein. The present lists of exosome proteins do not facilitate a detailed understanding of exosome biology. However, the biological significance and clinical utility of exosome proteins may be clearer when data will be associated with clinicopathological parameters.

Cells and secretions in the alveolar and bronchial air spaces can be recovered by bronchoalveolar lavage (BAL). BAL is a safe, reproducible, and minimally invasive procedure, and BAL proteins may be considered as a source in biomarker studies. Archimandriti et al. investigated BAL proteins from donors in the Metsovo area in Northwestern Greece (Metsovites) [85], where people have been exposed to asbestos due to the use of tremolite-containing whitewash [86, 87]. Comparative studies by 2D-PAGE showed that inflammatory proteins were upregulated in Metsovites, suggesting generation of reactive oxygen species via cytotoxic and cell activation reactions after asbestos exposure. A comparison of pleural calcification-positive and -negative subjects showed that proteins involved in regulation of the cell cycle and cell death as well as detoxification of endogenous and exogenous mutagenic and toxic agents were upregulated in pleural calcification-negative subjects. These included acid ceramidase, GST, and calcyphosine. These proteins may be involved in an increased susceptibility to mesothelioma in Metsovites without pleural calcification.

The discovery of therapeutic targets is critical to improving the clinical outcome of MPM. Kinase inhibitors have been approved for cancer management and numerous others are under investigation. Menges et al. examined phosphotyrosine in an MPM cell line to identify hyperactivated tyrosine kinases and signaling pathways, which could include candidate therapeutic targets [88]. Phosphotyrosine proteins were purified with pan-phosphotyrosine antibody, separated by SDS-PAGE, and subjected to LC-MS/MS. Nine phosphotyrosine proteins were identified and validated by western blotting. In addition to EGFR and MET, which are receptor tyrosine kinases previously identified to be aberrantly expressed in MPM cells, the novel nonreceptor tyrosine kinases, SRC, FYN, FER, and JAK1, were identified in MPM cells. Moreover, one transcription factor (STAT1) and scaffolding proteins (cortactin and p130Cas) were found to be tyrosine-phosphorylated in MPM cells. These data suggest that multiple tyrosine kinases are coactivated in MPM cells, and hence, a single tyrosine kinase inhibitor may not be effective in treatment of MPM. These findings were supported by a previous report that single-agent erlotinib therapy had no efficacy in a phase II clinical trial involving patients with MPM [89]. Kuramitsu et al. also investigated therapeutic targets using a proteomic approach [90]. Overexpression of the astrocytic phosphoprotein PEA-15 was identified in MPM cell lines in a comparative study of normal pleural and MPM cell lines by 2D-PAGE. The suppression of PEA-15 by siRNA reduced cell proliferation suggested the possible utility of PEA-15 as a molecular target.

Proteomic studies of MPM have been conducted from multiple viewpoints with an aim of developing clinical applications. The experimental data from *in vitro* studies and a small number of patient samples should be confirmed using clinical materials from a larger number of patients associated with clinicopathological data. A public biobanking system for clinical materials from patients with MPM has been reported [91, 92]. It would be beneficial if such a public banking system could be used to validate proteomics results.

### 2.9. Malignant Peripheral Nerve Sheath Tumor (MPNST)

MPNSTs constitute approximately 5%–10% of all soft tissue sarcomas. It emerges from peripheral nerves or cells associated with the nerve sheaths, such as Schwann cells. There is a higher incidence in patients with a radiation exposure history [93], while approximately half of MPNSTs occur in patients with the tumor disposition syndrome neurofibromatosis 1 (NF1) [94, 95]. NF1 is also known as von Recklinghausen disease and is a common autosomal dominant disorder characterized by pigmented skin lesions (cafe-au-lait spots), tumors, and iris malformation. Prognosis of patients with MPNSTs is poor with an overall 5-year survival rate of 34% [96]. The NF1 gene encodes a protein known as neurofibromin; its loss may constitutively activate the RAS signaling pathway and contribute to tumor development [97].

Dasgupta et al. studied differences in global protein expression by NF1-deficient astrocytes [98]. 2D-PAGE studies showed that NF1-deficient astrocytes had higher expression levels of proteins related to cell growth, such as nucleophosmin, regulators of protein translation and downstream targets of mTOR signaling. *In vitro* experiments demonstrated that treatment with rapamycin, an inhibitor of the mTOR signaling pathway, reduced cellular proliferation. Patrakitkomjorn et al. investigated neurofibromin-associated proteins to reveal the functional roles of NF1 deficiencies [99]. C-terminal domains of neurofibromin, which have a crucial role in regulating neurofibromin function, were fused with GST and transfected into astrocytes. Proteomics using iTRAQ identified more than 58 neurofibromin-associated proteins including CRMP-2. CRMP-2 is known to be a key molecule in axon formation and guidance. 2D-PAGE demonstrated that NF1 siRNA treatment resulted in upregulation of a series of CRMP-2 phosphorylations, while specific kinase inhibitors affected CRMP-2 phosphorylation and morphological neuronal differentiation.

The global protein expression of cells showing over- or underexpression of target genes and identification of the proteins binding to the products of causal genes are a standard approach for studying diseases with common mutations. Two MPNST studies focused on NF1 and reported the aberrant activation of signal pathways due to NF1 deficiency and proteins binding to the NF1 product neurofilamin. Classic 2D-PAGE and iTRAQ were used to assess protein expression. However, these modalities cover only a limited proportion of the proteome, and hence, the use of other techniques may help to identify other interesting proteins.

### 2.10. Neuroblastoma (NB)

NB is the third most common malignant tumor in children. The clinical behavior of NB varies according to patient age and diagnosis. In patients older than 1 year of age, the tumor is aggressive and metastatic and patients have a poor long-term survival rate of approximately 30%. However, in patients under 1 year of age, the tumor frequently regresses or differentiates, and patients have a favorable prognosis [100].

To investigate the potential mechanisms of NB progression, Chen et al. used the isotope-coded affinity tag approach to examine the proteome of primary tumor tissues from patients at stage 4 MYCN-amplified NB and those at stage 1 MYCN-not amplified NB [101]. Of the 1461 proteins observed, 566 had differential expression in the two patient groups. Pathway analysis demonstrated that proteins involved in growth promotion, Myc targets, and proteins in stemness signatures were upregulated in stage 4 tumor sample. Network analysis showed that MYCN had a central role in regulating these proteins. It was noteworthy that protein and mRNA expression was moderately associated because approximately half of the upregulated proteins in stage 4 tumor tissues had increased mRNA levels while one-third of downregulated proteins had lower mRNA expression. Further studies on these candidate proteins and networks may contribute to target discovery.

Yu et al. investigated the proteomic backgrounds of spontaneous regression in NB [102]. They compared two types of tumor samples, that is, stage 4 and stage 4s. In stage 4 NB, the tumor spreads to distant organs, whereas the tumor shows localized growth or restricted metastasis in stage 4s NB. Patients in stage 4 often have MYNC amplification and a poor prognosis despite intensive therapy, while those in stage 4s often present with a high survival rate and spontaneous regression. A comparative study by 2D-DIGE identified nine proteins with different expression in stage 4 and stage 4s, which included proteins involved in the organization of differentiation, proliferation, and apoptosis. These proteins may be candidate biomarkers for predicting a favorable prognosis.

 A recent study suggested that relapse and progression in NB cannot be reliably detected by monitoring known biomarkers [103]. To investigate biomarkers of the malignant features of NB, Hsu et al. examined proteomic changes in NB cells induced by neuronal differentiation [104]. 2D-DIGE experiments identified 24 proteins that were associated with differentiation, and an association between GRP75 expression and favorable clinical outcome was confirmed in 72 patients with NB by immunohistochemistry. GRP75 was highly associated with *in vitro* and *in vivo* differentiation status of NB tumor expression, suggesting that it may negatively regulate the growth of NB. Zanini et al. compared NB and Ewing sarcoma using 2D-PAGE and found the unique expression of HSP27 in NB [105]. Expression of HSP27 was regulated by an all-*trans* retinoic acid (ATRA) treatment, and immunohistochemistry revealed an association of HSP27 with a favorable prognosis in patients with NB. The prognostic utility of GRP75 and HSP27 should be further validated in an independent cohort to establish its possible role as a tool for risk stratification in patients.

Plasma biomarkers for the early diagnosis and monitoring of disease progression were investigated using proteomic approaches [106–110]. Tumor markers that are commonly used for NB, that is, urinary catecholamines vanillylmandelic acid and homovanillic acid, were elevated in only 50% of patients at the time of relapse. Egler et al. examined plasma samples from 55 patients with NB using SELDI-ToF MS and those from 33 NB patients using a Luminex suspension bead array [110]. Peptides that were present at a higher level in low-grade NB were subjected to MS and literature mining, and apolipoprotein 1 (APOA1) and serum amyloid A (SAA) were identified as candidate proteins. Expression of APOA1 in low-grade NB and healthy donors was higher than that in high-grade NB and was confirmed in the same sample sets by ELISA. A Luminex bead array system detected five cytokines as biomarkers that discriminated high-grade NB from low-grade NB and healthy donors by measuring the area under the empirical receiver operating characteristic curves. SAA allowed significant discrimination performance in both ELISA and AUC analyses. Seven proteins discriminated most high-grade NBs from low-grade NBs in the two independent sample sets. Biomarkers of high-grade NBs were investigated using an animal model and 2D-PAGE [109]. Plasma samples were compared between patients with NB and healthy donors by SELDI-ToF MS [106, 108], while conditioned media from NB cell lines were also examined by 2D-PAGE [107].

Prediction of the effects of chemotherapy is critical for optimizing existing therapeutic tools. Biomarkers that predict chemosensitive and chemoresistant phenotypes were investigated using topoisomerase inhibitors [111], docetaxel, paclitaxel [112], and cisplatin [113] with proteomic approaches. D'Aguanno et al. compared the proteome of the NB cell line SH-SY5Y and its cisplatin-resistant counterpart using 2D-PAGE and label-free nLC-MS [113]. Literature mining of the identified proteins found 13 papers with representative transcriptomic and proteomic studies on cisplatin resistance, which suggested the activation of the transcription factor nuclear factor erythroid 2-related factor 2 (Nrf2) in cisplatin-resistant NBs. Nrf2 is a transcription factor that regulates a cellular protective response. Recent studies have suggested that Nrf2 inhibition may render cancer cells more susceptible to chemotherapeutic drugs such as cisplatin [114]. Therefore, proteins involved in the Nrf2 pathway may have clinical utility as predictive biomarkers and therapeutic targets.

There is a global need for biomarkers that monitor responses to chemotherapy. Proteomic approaches have been used to identify cellular proteins indicating the effects of a kinase inhibitor (Sorafenib) [115], anti-Hsp90 drug (17-*N*-Allylamino-17-demethoxygeldanamysin, 17-AAG) [116], extracts of medical plants [117, 118], ATRA [119], and an MAPK inhibitor (U0126) [120]. Various proteomic modalities such as 2D-PAGE, 2D-DIGE, GeLC-MS/MS, and SILAC have been used in global expression studies, leading to the identification of many proteins with changed expression after treatment. The results were confirmed by *in vitro* experiments, but all these studies were performed using cell lines only, and hence, clinical utility of the proteins identified remains unclear. It is difficult to obtain tumor tissue samples before and after treatment with antidrug reagents. However, many lines of evidence suggest the presence of tumor-derived proteins in the plasma and their utility as biomarkers [121]. Thus, it may be a good idea to examine the plasma levels of these proteins in patients who have received chemotherapy.

Cell lines and xenograft models may have a greater potential as tools for biomarker development and target discovery in NB. 2D-PAGE data were used to create a proteome map of xenografts [122], which identified protein expression in cell lines and xenografts [123]. The proteomes of cells grown in a three-dimensional cell culture system were compared with conventional monolayer cell models by 2D-PAGE [124].

### 2.11. Synovial Sarcoma

Synovial sarcoma is the third most common primary mesenchymal tumor and accounts for 5%–10% of all soft tissue sarcomas [125]. The clinical course of synovial sarcoma spans a wide spectrum, from a curable disorder to a highly malignant disease [126]. Synovial sarcoma is characterized by the presence of a fusion of the SYT gene with either SSX1 or SSX2, as a consequence of a chromosomal translocation [127]. The fusion type has diagnostic significance and prognostic value, and patients with SYT-SSX1 fusion tumors have a less favorable prognosis than those with SYT-SSX2 [128]. Synovial sarcoma is histologically classified into two major types—biphasic subtype, which composed of epithelial and spindle tumor cells, and monophasic subtype, which composed solely of spindle tumor cells. The molecular backgrounds of the histological subtypes and their association with clinical observations have been extensively studied [128], but no conclusive results have been obtained.

Suehara et al. investigated the protein content of primary tumors of synovial sarcoma patients with different prognoses with an aim of developing prognostic biomarkers [129]. A proteomic analysis using 2D-DIGE identified 29 proteins that had different expression levels in patients with metastasis within 2 years of surgical resection and those with no metastasis more than 5 years after surgery. The prognostic value of secernin-1 was further examined in 45 newly enrolled patients with synovial sarcoma. Using the original antibody against secernin-1, an immunohistochemical study showed that patients with secernin-1-positive primary tumors had a better prognosis than patients with secernin-1-negative primary tumors. Secernin-1 recruits secretory granules to the site of exocytosis leading to increased granule swelling, core expulsion, or breakdown in tumors. It is not clear how secernin-1 is involved in the malignant features of synovial sarcoma, but the association of secernin-1 with the prognosis seems to be significant, and it would be worthwhile continuing the validation of this study.

To address the molecular mechanisms involved in the histological subtypes of synovial sarcoma, Suehara et al. investigated tumor tissues with different histological subtypes [130]. 2D-DIGE experiments identified 29 proteins with differential expression in biphasic and monophasic synovial sarcomas, and the unique expression of GST-P1 in biphasic synovial sarcoma was further validated in an additional 42 cases by immunohistochemistry. GST-P1 functions in xenobiotic metabolism, and it has an important role in susceptibility to cancer and other diseases. Immunohistochemistry demonstrated that GST-P1 expression was almost exclusively in the epithelial components of biphasic subtypes and most monophasic subtypes, whereas the spindle components of biphasic subtypes were GST-P1-negative. GST-P1 expression was independent of any clinicopathological parameters, including fusion gene status. These results established GST-P1 as a novel histological biomarker, and it was suggested that GST-P1 may have an important role in the epithelial differentiation of synovial sarcoma.

These proteomic studies were based on clinical and histological observations, and proteomic approach successfully identified possible biomarker candidates for diagnostic and prognostic purposes. The association between the identified biomarker proteins and known specific biomarkers such as fusion gene products are of further interest. As is the case with other biomarker studies, the functional backgrounds of biomarker molecules remain unclear yet but it would be useful to investigate the functional properties of secernin-1 and GST-P1 in the malignant features of synovial sarcoma, in parallel with validation studies.

### 2.12. Clear Cell Sarcoma (CCS)

CCS is a rare melanin-producing soft tissue sarcoma [131] that is characterized by a recurrent chromosomal translocation, resulting in the fusion of the EWS and ATF1 genes [132]. CCS is a high-grade sarcoma with a poor overall survival [133], and despite intensive research, the only effective cure is surgical resection at an early stage [134, 135]. Thus, there is a need to establish effective therapeutic strategies to control the local disease and distant metastasis.

Cell lines and xenograft models can be used to investigate the molecular basis of tumors and develop novel biomarkers and therapeutic targets. They are particularly in demand for rare diseases such as CCS. Dimas et al. examined the proteomic profiles of two cell lines and corresponding xenografts from a patient with CCS using 2D-PAGE [136]. Approximately 1250 proteins were observed and 124 were common between the cell lines and primary xenografts, while the others were unique, including proteins in the C-MYC pathway, apoptosis-regulating proteins, oncogene and tumor-suppressor gene products, and those with stem cell characteristics.

 Cell line and xenograft models have great potential in biomarker studies and pharmacological assessments. However, Celis et al. reported that the expression level of certain proteins changed even after short-term cell culture of primary bladder cancer tumors [137]. Therefore, a comparison of primary tumor tissues, their corresponding cell lines, and xenografts will be important when evaluating the utility of model systems. This study demonstrated that many proteins were observed, exclusively in cell lines and xenografts, suggesting the need for criteria in the establishment and validation of preclinical models of CCS.

### 2.13. Alveolar Soft Part Sarcoma (ASPS)

ASPS is a rare tumor that generally occurs in soft tissues of the extremities of young adults [138]. ASPS diagnostic histology includes a nest-like or organoid pattern separated by fibrovascular septa [138]. However, diagnosis of ASPS in an unusual location, such as the lung, stomach, retroperitoneum, and female genital tract may be difficult because a number of more common tumors mimic the histological features of ASPS [139]. Distinctive periodic acid-Schiff-positive crystals are another classical histological feature of ASPS [140], but typical crystals are observed only in a limited number of ASPS cases [138]. The detection of an ASPSCR1-TFE3 fusion transcript by RT-PCR is considered to be a marker of ASPS, but novel markers for immunohistochemical study are also required [141].

Balgley et al. compared the protein content of ASPS with uterine leiomyoma to identify a diagnostic protein biomarker for ASPS [142]. This study also aimed to assess the potential of formalin-fixed and paraffin-embedded (FFPE) tissue specimens for proteomic studies after long-term storage for several decades. Proteins were extracted from FFPE tissue specimens of tumors, digested with trypsin, and examined by combined capillary isoelectric focusing/nanoreversed phase liquid chromatography separation coupled with electrospray ionization—MS. Of the 2583 proteins identified, 80 were uniquely observed in ASPS. Immunohistochemistry confirmed the unique expression of vacuolar proton translocating ATPase 116 kDa subunit isoform a3.

It was interesting that proteomic study was feasible for samples that had been stored for several decades as FFPE. The application of FFPE to cancer proteomics has been examined for other malignancies, including ASPS. ASPS is particularly rare compared with other soft tissue sarcomas, and hence, the use of FFPE would be more valuable in ASPS. The diagnostic value of vacuolar proton translocating ATPase 116 kDa subunit isoform a3 should be validated with a large sample set, in parallel with functional assessments. Separation by capillary isoelectric focusing prior to LC-MS/MS was also a unique point of this study.

## 3. Overviews of Sarcoma Proteomics

### 3.1. Biomarker Study

Biomarker discovery is one of the major themes in soft tissue sarcoma proteomics, and surgical specimens and body fluids have been used to acquire clinicopathological data. Various types of clinical materials have been used for soft tissue sarcoma proteomics, such as surgical specimens, serum, and pleural effusions. In case of GIST, proteomics identified a prognostic biomarker using surgical specimens, and the results were successfully confirmed by multi-institutional validation studies with conventional immunohistochemistry [28, 31, 32]. The number of patients with soft tissue sarcoma is relatively small, and hence, validation is particularly difficult. Certain soft tissue sarcomas have common genetic abnormalities, and biomarker studies of such tumors may be promising even when studies begin with a limited number of samples. Functional studies may support the validity of biomarker candidates.

### 3.2. Therapeutic Target Identification

Identification of therapeutic targets is one of most important areas in cancer research. In particular, the global investigation of aberrant kinases may have bright prospects because many examples of small molecules, and specific antibodies that are directed against kinases have been previously reported. Conventional proteomics methods such as 2D-DIGE and MS may not be suitable for surveying global kinase expression because their expression levels are below the detection limit. The subjects of proteomic studies are defined before analysis; hence, antibody-based proteomics may be more effective than other methods. The use of antibodies against kinases and proteins on arrayed slides allows analysis of kinases in signal transduction pathways, which may not be achieved otherwise [53]. Similar approaches should be considered for other types of soft tissue sarcomas. In general, it may be useful to focus on proteins with known inhibitors and to examine their expression using specific antibodies.

### 3.3. Disease Mechanisms

Disease mechanisms are of general concern in cancer research because they can pave the way for novel discovery and clinical applications. A simple comparison of diseased samples and their normal counterparts could generate many differences depending on the characteristics of the proteomics method employed. More focused approaches, such as those on fusion gene products or etiologic virus proteins, may be a more efficient means of understanding disease mechanisms. If clinical materials are used in proteomic studies, proteome data should be associated with well-organized clinicopathological parameters from a statistically significant number of cases, and proteomic results should be validated by *in vitro* studies. A good example of a disease mechanism study was performed by Yang et al. They provided interesting insights into MErT in leiomyosarcoma using RPA and proposed a regulatory role for Slug on E-cadherin in MErT, thereby supporting their hypothesis by *in vitro* studies while they demonstrated clinical significance by TMA [42].

### 3.4. Proteomic Methods

All the major conventional proteomic methods have been used in soft tissue sarcoma proteomics, including 2D-PAGE, reverse protein array, and MS. In tumors where pathogenic gene alterations were identified, a focused approach using MS and antibodies appeared to be effective for surveying the results of such aberrations, while many proteins associated with aberrant gene products were identified by MS and Y2H. Antibody-based proteomics also provided novel findings based on the assumptions of existing biology. In contrast, expression studies were undertaken to observe global protein expression and many unexpected results were obtained by 2D-PAGE and MS. The simultaneous use of these two approaches should be considered in comprehensive studies.

Some front-end applications that are available for MS have not been employed in analysis of soft tissue sarcomas such as tissue imaging and selected reaction monitoring (SRM). The advantage of tissue imaging by MS has been reported for differential diagnosis [143], and a similar approach could be taken for soft tissue sarcomas. Quantitative proteomics by SRM may also be useful for biomarker development and target discovery in disease proteomics [144], which could also prove useful for soft tissue sarcoma analysis.

## 4. Problems and Perspectives in Soft Tissue Sarcoma Proteomics

There are two major inherent limitations in soft tissue sarcoma proteomics. First, the number of patients included in studies is generally insufficient to generate conclusive results. In the case of sarcomas with a homogeneous molecular etiology, such as those with common fusion genes or mutations, the use of a small number of samples during the initial stage could be compromised in a subsequent extensive validation study. Multi-institutional collaboration and biobanking system are required for validation because of the low frequency of soft tissue sarcomas. Second, many different proteomics methods have been applied to soft tissue sarcomas, but no single sarcomas have been exhaustively studied using multiple proteomics approaches. Individual proteomics methods visualize different aspects of the proteome with some overlaps; thereafter, the use of multiple methods and the integrations of these results should be considered to provide a more comprehensive understanding of the proteomic backgrounds of soft tissue sarcomas.

These two limitations are more or less true for proteomic studies with any type of malignancy and these issues are shared in the proteomics community. Efforts to establish a public database for disease proteomics [145] and to construct local, regional, national, and international biobanking systems [91, 92, 146] will facilitate soft tissue sarcoma proteomics. The standardization of proteomics data will contribute to the integration and sharing of proteomics data on soft tissue sarcomas [147].
